# Effect of photodynamic therapy (PDT) on *Enterococcus faecalis* biofilm in experimental primary and secondary endodontic infections

**DOI:** 10.1186/1472-6831-14-132

**Published:** 2014-11-04

**Authors:** Christian Tennert, Katharina Feldmann, Edwina Haamann, Ali Al-Ahmad, Marie Follo, Karl-Thomas Wrbas, Elmar Hellwig, Markus J Altenburger

**Affiliations:** Department of Operative Dentistry and Periodontology, University of Freiburg – Medical Center, Hugstetter Str. 55, 79106 Freiburg, Germany; Department of Hematology and Oncology, Core Facility, University of Freiburg – Medical Center, Hugstetter Str. 55, 79106 Freiburg, Germany

**Keywords:** Photodynamic therapy, PDT, Photoactivated chemotherapy, Phototherapy, Laser, Root canal disinfection, Endodontic infection, Enterococcus faecalis

## Abstract

**Background:**

To determine the antibacterial effect of photodynamic Therapy on *Enterococcus faecalis (E. faecalis)* biofilms in experimentally infected human root canals in primary infections and endodontic retreatments.

**Methods:**

One hundred and sixty single-rooted extracted teeth with one root canal were prepared using ProTaper instruments. Seventy specimens were left without root canal filling and autoclaved. The root canals of another 70 specimens were filled with Thermafil and AH Plus and the root canal fillings were removed after 24 hours using ProTaper D files and plasma sterilized. The specimens were infected with a clinical isolate of *E. faecalis* for 72 hours. Samples were taken using sterile paper points to determine the presence of *E. faecalis* in the root canals. The specimens were randomly divided into groups according to their treatment with 20 teeth each and a control. In the PDT group the teeth were treated using PDT, consisting of the photosensitizer toluidine blue and the PDT light source at 635 nm. In the NaOCl (sodium hypochlorite) group the root canals were rinsed with 10 mL of 3% NaOCl. In the NaOCl-PDT group the root canals were rinsed with 10 mL of 3% of sodium hypochlorite and then treated with PDT. Samples were taken after treatments using sterile paper points. Additionally, remaining root canal filling material was recovered from the root canal walls. Survival fractions of the samples were calculated by counting colony-forming units. A one-way analysis of variance (ANOVA) was applied to the data to assess the effect of different treatment techniques.

**Results:**

Antimicrobial treatment of root canals caused a significant reduction of bacterial load in all groups. NaOCl irrigation eliminated *E. faecalis* most effectively. PDT alone was less effective compared to NaOCl irrigation and the combination of NaOCl irrigation and PDT. CFU levels recovered from the filling material after NaOCl irrigation of the root canals were 10fold higher compared to PDT and the combination of NaOCl irrigation and PDT.

**Conclusions:**

Photodynamic therapy killed *E. faecalis* in experimental primary endodontic infections and retreated human root canals. PDT is an effective supplement in root canal disinfection, especially in endodontic retreatments.

## Background

A root canal treatment is the combination of mechanical instrumentation of a root canal system, its chemical debridement and filling with an inert material, designed to maintain or restore the health of the periradicular tissues. Although, infecting microorganisms are removed during mechanical debridement combined with chemical irrigation, residual bacteria are readily detectable in approximately 50% at the time of root canal filling, despite extensive irrigation with sodium hypochlorite [1]. Bacteria have been found in isthmi, lateral canals, fins, ramifications and anatomical structures, that remain inaccessible for mechanical instrumentation [2, 3].

Bacteria and bacterial toxins remaining in the root canal system after chemo-mechanical preparation or entering the filled root canal system via salivary microleakage, leakage of the coronal restoration or inadequate root canal filling may lead to failure [4–7]. Post-treatment disease associated with poor endodontic treatment may be caused as a result of not using rubber dam, poor access resulting in missed untreated root canals, inadequate instrumentation, inadequate disinfection, inadequate root canal filling or iatrogenic errors, e.g. fractured instrument, perforation, ledge [8].

Root canal retreatment is considered more difficult compared to primary root canal treatment as there are usually intracanal obstacles to overcome, e.g. gutta-percha removal, intracanal materials such as silver points, posts or fractured instruments, correcting iatrogenic errors such as ledges or perforations [8]. Previous studies have shown that root canal filling material cannot be removed completely leaving gutta-percha and/or sealer on the root canal wall making disinfection of the root canal system more difficult compared to primary root canal treatments [9–11].

After removal of root canal filling and other intracanal obstacles residual bacterial contamination has to be reduced to a minimum for successful endodontic treatment [12]. A favored method is alternating irrigation using sodium hypochlorite and a chelating agent, e.g. ethylenediaminetetraacetic acid or citric acid [13]. Chlorhexidine and iodine compounds have also been advocated as additional irrigants for root canal (re)treatment [14].

Endodontic failures are associated with high proportions of gram-positive aerobic and facultative microorganisms [15]. *Enterococcus feacalis*, which can be found in treated and untreated root canals, is highly associated with failures [16]. *Pseudomonas*, *Staphylococci* and *Streptococci* are also causative for failures.

Photodynamic therapy (PDT), also known as photoradiation therapy, phototherapy, photochemotherapy, or photoactivated chemotherapy (PACT) is a medical treatment, that utilizes the activation of a photosensitizing agent (photosensitizer) by exposure to light of a specific wave length in the presence of oxygen [17]. There is an energy transfer from the activated photosensitizer to available oxygen that results in the formation of toxic oxygen species, such as singlet oxygen and free radicals. Singlet oxygen and radical species cause a rapid and selective destruction of microorganisms. Most photosensitizers are activated by light between 630 and 700 nm. The main photosensitizers found in the literature are hematoporphyrin derivatives (620–650 nm), phenothiazine, like toluidine blue and methylene blue (620–700 nm), cyanine (600–805 nm), phytotherapic agents (550–700 nm) and hytalocyanines (660–700 nm) [18–20].

PDT has been shown to be an adjunctive therapy to conventional endodontic treatment to optimize the microbial reduction in root canals in primary endodontic infections [21–23]. Garcez et al. [24] investigated the effect of PDT in endodontic retreatments in vivo. They found that PDT as an adjuvant to conventional endodontic treatment leads to a significant further reduction of bacterial load after irrigation using sodium hypochlorite, hydrogen peroxide and EDTA and is effective against multi-drug resistant bacteria. In retreatments it is impossible to remove the root canal filling completely, impeding disinfection of the root canal system. Based on previous findings, PDT is supposed to have an additional antimicrobial effect after root canal irrigation, especially on resistant microorganisms.

The aim of the present study is to investigate the antibacterial effect of photodynamic Therapy (PDT) on *Enterococcus faecalis* biofilms in experimentally infected human root canals of primary and secondary endodontic infections.

## Methods

### Tooth specimens

For this study, we selected one hundred and sixty intact extracted permanent human front teeth and premolars. Two radiographs were taken of each specimen, one buccal-lingual/palatal and one mesial-distal image, to ensure that the specimens had normal pulp chambers, patent root canals and fully formed apices without any sign of resorption. Teeth with root canal fillings were excluded.

The Teeth were extracted at the Center for Dental Medicine, Department of Maxillofacial Surgery, University Medical Center Freiburg, because of acute tooth pain, severe inflammatory complications from systemic diseases, within the context of orthodontic treatment, acute infections (abscess), poor general health or, in the case of wisdom teeth, before complications from orthodontic treatment. Patients gave their written informed consent for using the extracted teeth for research, which was reviewed and approved by the ethics committee of the University of Freiburg (175/13).

Standard access cavities were prepared and the precise tooth length was determined by inserting an ISO 10 K-file (VDW®, Munich, Germany) into the canal until the file was visible at the apical foramen. The tip of the file was then placed exactly at the apical foramen of the tooth to measure the tooth length. Working length (WL) was set 1 mm short of tooth length. Root canals were prepared using ProTaper instruments (Dentsply®, Konstanz, Germany) according to the manufacturer’s instructions in combination with an Endo IT Professional (VDW®, Munich, Germany) at 250 rpm. During root canal preparation a 3% sodium hypochlorite solution was used for irrigation [25]. Root canal preparation was performed using ProTaper shaping files S1 and S2 and finishing files F1 and F2. Apical preparation was performed terminating with ProTaper F2 file at WL. A ProTaper F2 gutta-percha point was set at WL and the apical region was covered with composite using Optibond FL (Kerr Corporation, Orange, CA, USA) and CeramX mono (Dentsply®, Konstanz, Germany) for an apical seal. Teeth were embedded in methacrylate (Techovit 4070™, Haereus Kulzer®, Wernheim, Germany) and then decoronated using a diamond rotary cutting instrument (6830 L.314.014, Gebr. Brasseler GmbH & Co. KG, Lemgo, Germany) at 40.000 rpm at the cementum-enamel junction. After a final irrigation with 3% sodium hypochlorite and 5% sodium thiosulfate, the teeth were subsequently autoclaved at 134°C for 18 minutes. To verify that the root canals were free of microorganisms, samples were taken from the root canals using sterile ProTaper F2 paper points. Paper points incubated in the root canal for one minute and then transferred into a vial containing 750 μl of sterile Ringer’s solution, vortexed for 30 seconds and 100 μl were cultured on a blood agar plate for 24 hours. Teeth with a positive culture were excluded.

### Root canal filling and retreatment

The root canals of 70 specimens were filled using a size 25 Thermafil obturator (Dentsply®, Konstanz, Germany) in combination with an epoxy resin-based sealer (AH Plus, Dentsply®, Konstanz, Germany). For decontamination the Thermafil obturators were incubated in a vial with 3% sodium hypochlorite solution for 10 min [26]. The sealer was placed into the coronal third of the root canal with a sterile ProTaper F2 paper point. Each obturator was heated using a ThermaPrep Plus Oven (Dentsply®, Konstanz, Germany) until an audible signal indicated that the obturator was ready for placement. It was then inserted into the prepared root canal with a slow, firm and continuous movement in apical direction. The handle of each obturator was stabilized during cooling. The carrier was not cut to be able to remove it easily. 2 radiographs were taken from each specimen, one buccal-lingual/palatal and one mesial-distal image, to ensure complete filling of the root canal.

After 24 hours the carriers were removed and the root canal filling was removed using ProTaper D1 and D2 instruments until working length was reached using 5× magnification. After removal of the root canal filling 2 radiographs were taken from each specimen to visualize the remaining root canal filling material.

After removing the root canal filling the specimens were sectioned longitudinally using a rotary cutting diamond saw under water spray. Specimens were sterilized using plasma sterilization (H_2_O_2_). After sterilization the two sections of the specimens were attached to each other using an adhesive (Heliobond, Ivoclar Vivadent, Ellwangen, Germany).

### *Enterococcus faecalis*infection

Root canals were infected with a clinical isolate of *E. faecalis. E. faecalis* was cultured overnight in Tryptic Soy Broth (TSB) (Sigma-Aldrich, St. Louis, MO, USA) at 36°C at 5 to 10% CO_2_ and syringed into the prepared root canal using a 30 gauge irrigation needle. Root canals were infected with *E. faecalis* for 72 hours to allow biofilm formation. TSB was changed every 24 hours. After 72 hours the root canals were rinsed with 5 mL of sterile Ringer’s solution and a sampled using 3 sterile ProTaper F2 paper points (Dentsply®, Konstanz, Germany) to evaluate the presence of *E. faecalis* in the root canals. The paper points were transferred into 750 μl of sterile Ringer’s solution. The samples were cultured on blood agar to determine the presence of *E. faecalis* in the root canals. 10 specimens that were not root filled and 10 specimens with retreated root canals were left without infection with *E. faecalis* and served as a control group.

After 72 hours samples were taken using paper points and cultured on blood agar to verify, that non-infected root canals were free from microorganisms.

### Antimicrobial treatment of primary infections

Seventy specimens with primary infections were randomly divided into three groups. In the first group (PDT group), the root canals of 20 specimens were treated using PDT (PACT200, Cumdente, Tübingen, Germany), which consists of a light source at 635 nm in combination with the photosensitizer PACT-Fluid Endo, a toluidine blue solution at 13–15 mg/mL. PACT-Fluid Endo was brought into the root canal using a 30 gauge needle. After 60 seconds of incubation the PACT-Fluid Endo was activated using the PACT light source with the PACT Light Guide Endo tip, a 100 mW LED light source at 635 nm. The PACT Light Guide Endo tip was set as close to WL as possible and activated for 120 seconds according to the manufacturer’s instructions. Root canals were rinsed with 5 mL of Ringer’s solution to remove the PACT-Fluid Endo solution, and sampled with 3 sterile ProTaper F2 paper points. Paper points were transferred into 750 μl of sterile Ringer’s solution, vortexed for 30 seconds and 100 μl were cultured on a blood agar plate and colony forming units were counted.

In the second group (NaOCl group), 20 root canals were irrigated with 10 mL of 3% NaOCl with a flow rate of 3–3.5 ml per minute. NaOCl was removed by rinsing the root canals with 2.5 mL of sterile Ringer’s solution.

In the third group, 20 root canals were treated with sodium hypochlorite and PDT (NaOCl-PDT group). The root canals were rinsed with 10 mL of 3% sodium hypochlorite using a 30 gauge irrigation needle with a flow rate of 3–3.5 ml per minute. Excess sodium hypochlorite was removed by irrigating with 2.5 mL of sterile Ringer’s solution and the root canals were treated using PDT and sampled according to the protocol of the PDT group. In a control group, 10 root canals were rinsed with 10 mL of Ringer’s solution.

### Antimicrobial treatment of secondary infections

For antimicrobial treatment of the secondary endodontic infections, 70 specimens were randomly divided into three groups. In the first group (PDT group), 20 root canals were treated using PDT (PACT, Cumdente, Tübingen, Germany) and sampled according to treatment of the PDT group of the primary infections.

In the second group (NaOCl group), 20 root canals were irrigated with 10 mL of 3% NaOCl with a flow rate of 3–3.5 ml per minute. NaOCl was removed by rinsing the root canals with 2.5 mL of sterile Ringer’s solution.

In the third group (NaOCl-PDT group), 20 root canals were rinsed with 10 mL of 3% sodium hypochlorite (NaOCl) using a 30 gauge irrigation needle with a flow rate of 3–3.5 ml per minute. NaOCl was removed by irrigation with 2.5 mL of sterile Ringer’s solution and a sample was taken using 3 sterile ProTaper F2 paper points. Afterwards root canals were treated using PDT and sampled as described above.

In the control group, 10 root canals were rinsed with 10 mL of Ringer’s solution.

After antimicrobial treatment samples were taken from all specimens using 3 sterile ProTaper F2 paper points and transferred into a vial containing 750 μl of sterile Ringer’s solution to determine the survival fractions.

Specimens were separated again using a sterile scalpel and the remaining root canal filling on the root canal walls was collected using a sterile handfile (VDW®, Munich, Germany) into a vial containing 5 mL of TSB and cultured overnight. 100 μl of the samples were then cultured on blood agar. Survival fractions of the samples were calculated by counting colony-forming units.

### Scanning electron microscopy (SEM)

For SEM examination tooth specimens, samples of Thermafil gutta-percha and AH Plus were prepared. Samples of freshly mixed AH Plus were set for 24 hours. AH Plus samples and Thermafil samples were disinfected using 70% ethanol. The tooth specimens were cut vertically in two pieces using a rotary cutting diamond saw. Tooth specimen sections were autoclaved at 134°C for 18 minutes. The tooth specimen sections and material samples were infected with *E. faecalis* in TSB for 72 hours in chambered coverslips (μ-Slide 8 well, ibidi GmbH, Martinsried, Germany). The material samples and tooth specimens were divided in three groups. The wells of group one did not get any antimicrobial treatment and served as a control. In group two the specimens and material samples were treated with PACT-Fluid Endo for 1 minute and the PACT Light source for 1 minute. In group three the specimens and material samples were incubated with 3% NaOCl for 1 minute. After antimicrobial treatment, the wells were rinsed with 1 mL of sterile Ringer’s solution. The samples and specimens were fixed in 8% formaldehyde overnight at 4°C and dehydrated in graded alcohol (30%, 50%, 70%, 80%, 90% once each and twice in 99.8% for 1 hour). Then critical point drying (Critical Point Dryer CPD 030; Bal-Tec, Wallruf, Germany) with liquid carbon dioxide was carried out according to standard procedure. The samples were sputtered with gold in an SCD 050 coater (Bal-Tec) and examined by using a Zeiss Leo 435 VP scanning electron microscope (Leo Electron Microscopy Ltd Cooperation Zeiss Leica, Cambridge, England) at 10–12 kV.

### Statistical analysis

A one-way analysis of variance (ANOVA) was applied to the data in order to assess the effect of different treatment techniques on the reduction of *E. faecalis* in root canals. The significance level was set to *p <0.05*. All statistical tests were conducted using SSPS (15.0).

## Results

### Effect of antimicrobial treatments in primary infections

**Figure 1:**
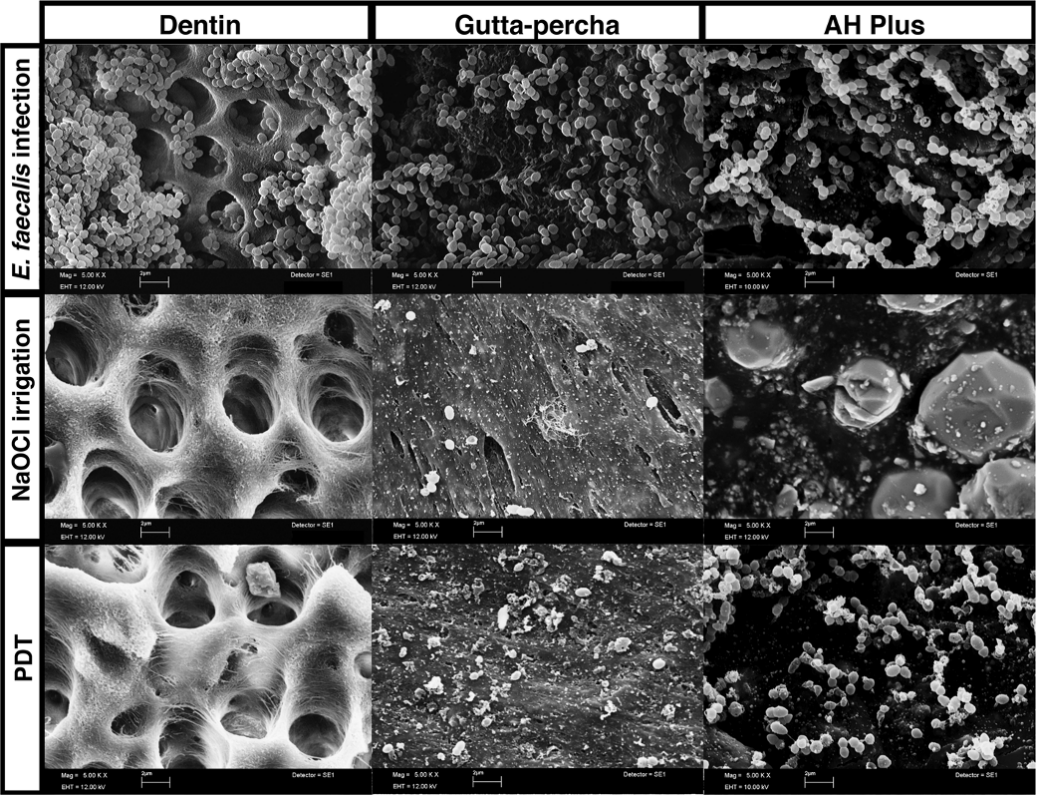
**Scanning electron micrographs of root canal dentin and the root filling materials Thermafil gutta-percha and AH Plus colonized with** ***E.faecalis*** **at magnification of 5000×.** Treatments using 3% NaOCl or PDT severely reduce *E. faecalis* on dentin and the surfaces of the root canal filling materials. There were found few normal shaped bacterial cells on gutta-percha and AH Plus after treatments. Remaining cells on dentin after treatments showed abnormal shape.

Figure 1 shows 72 hours old *E. faecalis* biofilm. Mean *E. faecalis* counts before antimicrobial treatments were 2,32x10^6^ CFU/ml.

Treatment of root canals with PDT alone caused a reduction in bacterial load, resulting in a 92.7% kill of *E. faecalis*. Rinsing root canals with 3% sodium hypochlorite (NaOCl) achieved a reduction of 99.9% and the combination of NaOCl disinfection and PDT reduced bacterial viability by 99.9%. Irrigation with 3% sodium hypochlorite and the combination of NaOCl irrigation and PDT led to a significantly higher reduction of *E. faecalis* compared to PDT treatment alone (p <0.0001). cases After irrigation with 3% NaOCl, 80% of the root canals were culture negative canals. The additional treatment using PDT increased the number of culture negative canals to 90%. When root canals were treated with PDT alone, 1 out of 20 cases (5%) was culture negative (Table 1).

**Table 1:** **Reduction of** ***E. faecalis*** **after antimicrobial treatment**

	Contamination with ***E. faecalis***CFU/ml × 10^6^		Treatment		Contamination after treatment CFU/ml × 10^6^		Reduction of ***E. faecalis***[%]	Culture negative samples [%]
	Mean	Std Dev	3% NaOCl	PDT	Mean	Std Dev		
Primary infection	2.48	2.04	X	-	0.0006	0.0003	99.9^a^	80
	2.22	1.92	-	X	0.16	0.14	92.7^b^	5
	2.13	1.86	X	X	0.0004	0.0003	99.9^a^	90
Secondary infection	2.24	1.76	X	-	0.0005	0.0001	99.9^a^	55
	2.57	2.21	-	X	0.02	0.04	99.9^a^	0
	2.26	1.58	X	X	0.001	0.001	99.9^a^	30

Percentage of culture negative samples. Superscript letters indicate statistical differences.

### Effect of antimicrobial treatments in secondary infections

Antimicrobial treatment of the retreated root canals caused a significant reduction of bacterial load in all three groups, resulting in a 99.9% kill of *E. faecalis* (Table 1). NaOCl irrigation achieved culture-negative root canals in 55% of the cases, whereas in teeth treated with PDT no specimen was culture-negative. For the combination of NaOCl irrigation and PDT 30% of the cases were found culture-negative.

### *E. faecalis*isolated from the root canal filling material

Figure 1 shows 72 hours old *E. facealis* biofilm on Thermafil gutta-percha and AH Plus. In all groups *E. facealis* could be cultured from the isolated filling material recovered from the root canal walls. There were equal amounts of CFU recovered from root filling material specimens with PDT and the combination of NaOCl irrigation followed by PDT. CFU levels recovered from the filling material after NaOCl irrigation of the root canals were about 10fold higher compared to PDT and the combination of NaOCl irrigation and PDT.

## Discussion

In the present study, PDT causes a reduction of bacterial viability in both primary endodontic infection and retreated root canals infected with *E. faecalis*. Treating root canals with sodium hypochlorite (NaOCl), PDT or a combination of NaOCl irrigation and PDT caused a significant reduction of *E. faecalis* in the root canals. In the cases of primary infections, the combination of NaOCl irrigation and PDT NaOCl irrigation achieved the highest number of culture-negative root canals (90%), NaOCl irrigation alone achieved culture-negative root canals in 80%, whereas after treatment using PDT only one specimen was culture-negative. For secondary infections, NaOCl irrigation achieved the highest number of culture-negative root canals, whereas after treatment using PDT all the specimens were culture-positive. Garcez et al. [24] investigated the effect of PDT in endodontic retreatments in vivo. They found that PDT as an adjuvant to conventional endodontic treatment leads to a significant further reduction of bacterial load after irrigation using NaOCl, hydrogen peroxide and EDTA and is effective against multi-drug resistant bacteria. Based on these findings, PDT is supposed to have an additional antimicrobial effect after root canal irrigation, especially on resistant microorganisms. The results of the present study cannot confirm these findings in the cases of retreatments. Microorganisms might be able to invade dentin and dentinal tubules covered by root canal filling material and might not be accessible for disinfecting agents or PDT components. Also, irrigularities in the shape of root canals, fins and dentinal tubules could limit diffusion of NaOCl or the photosensitizer, oxygen and light. The remaining root canal filling material may harbour microorganisms making it impossible for irrigation solutions, the photosensitizer and the light to penetrate. The emitted light might be absorbed or reflected by residue root canal filling material limiting the effect of PDT. However, in the present study the isolated root canal filling material shows lower contamination with *E. faecalis* after treatment with PDT and the combination of NaOCl irrigation and PDT compared to NaOCl irrigation. The photosensitizer might have penetrated these areas of adherent filling material to the root canal wall better than NaOCl or NaOCl is inactivated when it gets in contact with *E.faecalis* or its biofilm-like structure components [27].

The resistance of microorganisms organized in biofilms has been described previously [28]. Bacteria in deep layers of the biofilm are more resistant due to the limitation of diffusion (photosensitizer, oxygen or light or the irrigating agent) through the organic structure [29]. Furthermore, organic compounds, such as dead cells or biofilm extracellular matrix, neutralize antimicrobial agents. Previous studies suggested to use PDT as an adjuvant treatment to irrigation in root canal disinfection [21–23, 30]. The authors found irrigation protocols based on NaOCl, hydrogen peroxide and chelating agents, like EDTA, to be superior in endodontic antimicrobial treatment, especially in terms of biofilm disintegration. However, Garcez et al. [30] has demonstrated an additional effect of PDT after conventional treatment and found *E. faecalis* to be 100–1000 times more sensitive to PDT mediated killing compared to Gram-negative species [31]. The lower antimicrobial effect of PDT compared to irrigation can be explained by dead cells and cell remnants that remain in the root canal, on the root canal wall or may still be integrated in the biofilm-like structure neutralizing the PDT mediated killing, so that the photosensitizer, oxygen and the light may not reach bacteria in deep layers. Irrigation removes the dead cells and remnants.

In the present study, a monospecies infection model with a clinical isolate of *E. faecalis* was used. *E. faecalis* has frequently been found in primary and most importantly secondary infections of root canal systems [32, 33]. *E. faecalis* has been shown to be very resistant against disinfecting agents and antibiotics [32]. *E. faecalis* can effectively be colonized, forms biofilm on root canal walls and invades dentinal tubules [34–36]. This monoinfection model was used to reproduce the same biofilm-like structure in each root canal of the specimens with a species that is known to be hard to eliminate by chemo-mechanical debridement [32]. However, root canal infections are often associated with multiple species [27, 37]. A biofilm with multiple species may differ in the content of the microorganisms in each specimen that might impair the effect of disinfection [27].

Samples from the root canals were taken using sterile ProTaper F2 paper points. Paper points only take a portion of bacteria from the root canal wall. This method of sampling will underestimate the CFU levels in the root canals before and after treatment and does not give the total *E. faecalis* counts for each specimen. A culture-negative sample does not mean a sterile root canal. For all samples ProTaper F2 paper points were used. These paper points have the same surface size and perfectly fit into the prepared root canal for sampling and this method has been used in previous studies [24, 29, 32]. Molecular techniques, like PCR techniques, are often used to detect microorganisms in root canals [37, 38]. But in the present study, it was important to differentiate between living (culturable) and dead (not culturable) bacteria. Molecular techniques would have led to false positive results when cell remnants and DNA are left in the root canal after antimicrobial treatment, overestimating the remaining contamination of the root canal. This overestimation would have been higher for specimens treated with PDT compared to irrigation, because irrigation will remove a lot of dead cells and cell remnants. Therefore, a second sample was taken after antimicrobial treatment using a sterile K-file to remove dentin chips from the root canal wall. In this way, the remaining contamination of the root canal wall and dentinal tubules was assessed. This limits the underestimation of the bacterial contamination by sampling using paper points.

Souza et al. [29] infected root canals before shaping. Instrumentation and irrigation procedures cause extra stress on microorganisms and disrupt the biofilm. It has been shown that instrumentation of root canals significantly reduces bacteria in root canals [39, 40]. In the present study, PDT has been tested in completely shaped root canals and in the case of endodontic retreatment after root canal filling and removal of the root canal filling. This does not imitate the clinical situation. These specimens were then infected with a clinical isolate of *E. faecalis*. Within 72 hours of infection, *E. faecalis* was able to form a biofilm-like structure and the effect of the different antimicrobial treatments on *E. faecalis* in root canals could be investigated.

## Conclusion

PDT reduces *E. faecalis* in infected primary infections and retreated root canals. However, irrigation with 3% sodium hypochlorite is more effective in eliminating *E. faecalis* compared to PDT. PDT was able to eliminate *E. faecalis* from remaining root canal filling material on the root canal wall more effectively compared to 3% NaOCl. PDT is not an alternative but an effective supplement in root canal disinfection, especially in cases of retreatments. Disruption of the biofilm prior to PDT and the adjunct use of a sodium hypochlorite based conventional approach remain mandatory.
